# Millennial-scale glacial climate variability in Southeastern Alaska follows Dansgaard-Oeschger cyclicity

**DOI:** 10.1038/s41598-019-44231-1

**Published:** 2019-05-27

**Authors:** Paul S. Wilcox, Jeffrey A. Dorale, James F. Baichtal, Christoph Spötl, Sarah J. Fowell, R. Lawrence Edwards, Johanna L. Kovarik

**Affiliations:** 1 Geoscience Department, University of Alaska Fairbanks, Fairbanks, AK 99775 USA; 2 Institute of Geology, University of Innsbruck, 6020 Innsbruck, Austria; 3 Department of Earth and Environmental Sciences, University of Iowa, Iowa City, IA 52252 USA; 4 Forest Service, Tongass National Forest, Thorne Bay, AK 99919 USA; 5 Department of Earth Sciences, University of Minnesota, Minneapolis, MN 55455 USA; 6 Forest Service, Mineral and Geology Management, Denver, 80401 Colorado USA

**Keywords:** Palaeoclimate, Geochemistry

## Abstract

A stalagmite from Prince of Wales Island grew episodically between ~75,000 and ~11,100 yr BP; interrupted by seven hiatuses. Hiatuses most likely correspond to permafrost development and a temperature drop of up to 5 °C from modern conditions. Intervals of calcite deposition place tight constraints on the timing of mild climatic episodes in Alaska during the last glacial period, when permafrost was absent, allowing water infiltration into the karst system. These periods of calcite deposition are synchronous, within dating uncertainties, with Greenland Interstadials 1, 10, 11, 12c, 14b-14e, 16.1a, 17.2, and 20c.

## Introduction

During the last glacial period the many regions experienced large variations in climate that were first identified in ice cores from central Greenland^1–3^. Known as Dansgaard-Oeschger (D*-*O) cycles, these climate oscillations have subsequently been recognized across the globe in places such as the monsoon regions of Asia^4,5^, parts of the Southern Hemisphere^6–8^, and the North Pacific^9–13^. The mechanisms of these abrupt warmings and intervening coolings (including ice-rafting events) remain a heavily researched topic in the paleoclimate community^14–16^.

Some sediment cores from the North Pacific have a sufficiently high resolution and extend back far enough to provide evidence of D*-*O cycles^9–13^, which are most prominent beyond 30,000 yr BP. In some cases there is good agreement between these marine proxy data and the Greenland ice-core data (e.g.^9,12^), supporting the idea of a teleconnection between the North Atlantic realm and the North Pacific. However, lack of suitable terrestrial samples at higher latitudes (>55°N) in the Pacific realm prevent this comparison. A robust and independent chronology of D*-*O cycles at high-latitude sites in North Pacific is therefore needed to further test the presence of teleconnections between these regions during the last glacial period. Here we present a speleothem record from Alaska that covers much of Marine Isotope Stage (MIS) 3, and provides a precise and independent chronology of short-lived warm intervals in Alaska. The robust dating of the speleothem record allows for a rigorous test of the temporal relationship of the observed warm phases in Southeastern Alaska with the D*-*O cycles observed in the North Atlantic.

## Site Location

**Figure 1:**
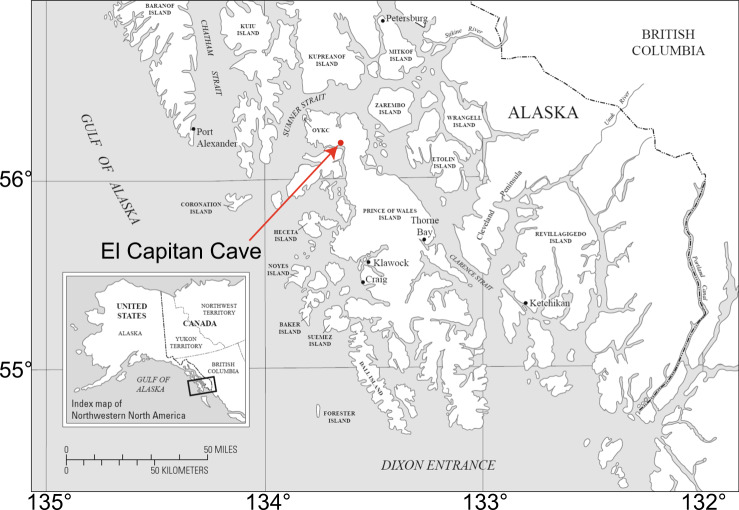
Map of southeast Alaska showing the location of El Capitan Cave.

Speleothem EC-16-5-F was collected from El Capitan Cave (56°9.72′N, 133°19.14′W) on Prince of Wales Island, Southeastern Alaska (Fig. 1). The cave is developed in the Silurian Heceta Limestone, a nearly pure carbonate unit^17^. El Capitan Cave is the largest known cave in Alaska, with a total surveyed passage length of 3.85 km and a mapped vertical extent of 131 m, consisting of three main levels. Speleothem EC-16-5-F was found 90 m inside the middle level, which is horizontal from the main cave entrance. The stalagmite is 14 cm tall and its upper (youngest) part was missing. This passage is ventilated only when a sump deeper in the cave is low enough to allow air flow, which typically occurs in summer. The interior of the cave where the stalagmite was collected is ~80 m above sea level (a.s.l), with the catchment above the cave reaching an elevation of ~700 m a.s.l.

## Climate and Vegetation

Prince of Wales Island has a maritime climate characterized by cool, generally wet conditions. Mean annual temperature and precipitation from Port Alexander, Alaska, 80 km northwest of El Capitan Cave, are 7.3 °C and 4115 mm, respectively (1981*–*2010^18^). The Gulf of Alaska is the main source of precipitation in the region, with storms generally associated with the semi-permanent Aleutian low-pressure system, especially in winter^19^. Mean annual air temperature (MAAT) in the interior of El Capitan Cave is approximately 5 °C, and is most likely a good approximation of MAAT at the cave site.

Most of Prince of Wales Island is covered by Pacific coastal rainforest interspersed with muskeg (peatlands with bog and fen communities)^20^, containing only C_3_ vegetation. Modern tree taxa in the region include *Picea sitchensis* (Sitka Spruce), *Tsuga heterophylla* (Western Hemlock), *Tsuga mertensiana* (Mountain Hemlock), *Alnus* (alder), and *Pinus contorta* ssp. *contorta* (Shore Pine)^21^.

## Results

### Petrography

**Figure 2:**
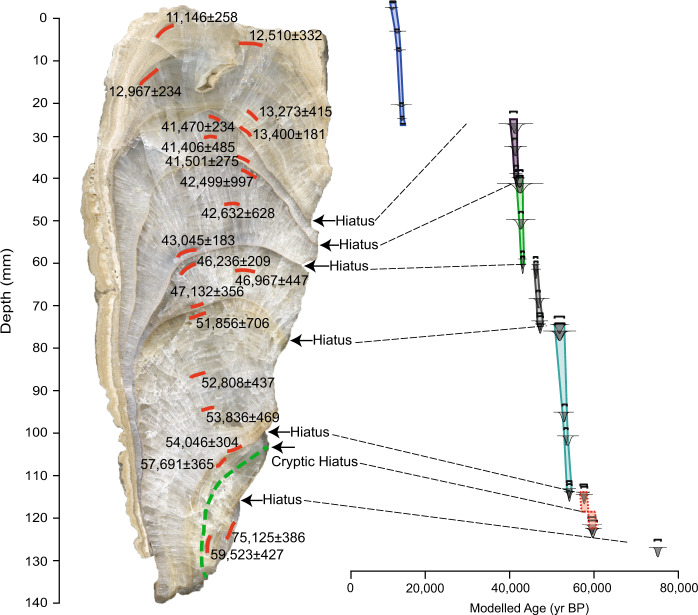
Polished slab of speleothem EC-16-5-F showing U-Th sampling locations and petrographic hiatuses. Note the presence of a cryptic hiatus (see text) represented by the dashed green line. Also shown is the time-depth model using OxCal 4.3. Note that the cryptic hiatus is incorporated into the age model (red boxes with dashed lines).

Speleothem EC-16-5-F is composed of a translucent compact-columnar calcite fabric (Suppl. 1). Crystal length and width range from 0.5 to 6 mm and 0.6 to 2 mm, respectively. Individual crystals show even extinction under cross-polarized light and contain few solid and liquid inclusions. The stalagmite is segmented by distinct petrographic boundaries at 27.4, 42.5, 65, 80.9, 124.3, and 133.9 mm (Fig. 2). Crystal growth came to a halt at four of the hiatuses. In the other two cases large columnar crystals continued to grow in optical continuity across the hiatus (Suppl. 1). All hiatuses are marked by a 5 to 100 µm thin layer of micrite. Only the hiatus at 27.4 mm shows indication of slight corrosion (Suppl. 1). There is no petrographic evidence of detrital sediment present at these hiatuses, which likely rules out flooding of the cave as a cause for these interruptions of growth. Most parts of this stalagmite reveal regular A-B-type epifluorescence banding, which most likely reflects seasonally-controlled influx of soil-derived fulvic and humic acids into the epikarst.

### Chronology

The 21 ages (Table 1, Fig. 2) define eight pulses of growth, separated by hiatuses corresponding to the petrographic boundaries (Fig. 2). Initial growth commenced at ~75,125 ± 386 yr BP based on a U-Th sample from the base of the speleothem. Because this segment is only 6 mm thick, only one date was obtained. Assuming a growth rate similar to the younger parts of this speleothem (i.e., ~0.02 mm/yr) it likely represents <500 yr of growth. Based on estimated ages from the time-depth model (Fig. 2), calcite deposition re-commenced at 59,750 ± 421 yr BP and lasted until 57,596 ± 797 yr BP. Subsequent growth phases estimated from the time-depth model are recorded between 54,147 ± 576 and 51,810 ± 1149 yr BP, 47,244 ± 669 and 46,165 ± 453 yr BP, 43,102 ± 374 and 42,339 ± 781 yr BP, 41,603 ± 501 and 40,972 ± 831 yr BP, and between 13,602 ± 459 and 11,100 ± 598 yr BP (Fig. 2). The growth period between 13,602 ± 459 and 11,100 ± 598 yr BP records the Younger Dryas interval.

**Table 1:** U-Th data from speleothem EC-16-5-F from El Capitan Cave.

Sample	^238^U	^232^Th	^230^Th/^232^Th	δ^234^U^*^	^230^Th/^238^U	^230^Th Age (yr)	^230^Th Age (yr)	δ^234^U_Initial_^Ɨ^	^230^Th Age (yr BP)***
Number	(ppb)	(ppt)	(atomic x10^−6^)	(measured)	(activity)	(uncorrected)	(corrected)	(corrected)	(corrected)
EC-16-5-F-5	111.4 ± 0.1	1860 ± 37	138 ± 3	378.9 ± 2.3	0.1393 ± 0.0008	11562 ± 73	11212 ± 258	391 ± 2	11146 ± 258
EC-16-5-F-7d	84.1 ± 0.1	1014 ± 21	209 ± 6	372.1 ± 2.5	0.1531 ± 0.0032	12831 ± 280	12577 ± 332	386 ± 3	12510 ± 332
EC-16-5-F-10	167.3 ± 0.3	2432 ± 49	183 ± 4	393.5 ± 2.1	0.1612 ± 0.0011	13334 ± 97	13034 ± 234	408 ± 2	12967 ± 234
EC-16-5-F-24c	85.5 ± 0.1	975 ± 20	244 ± 6	405.8 ± 2.3	0.1688 ± 0.0021	13866 ± 185	13340 ± 415	421 ± 2	13273 ± 415
EC-16-5-F-28	134.0 ± 0.1	1204 ± 24	305 ± 7	406.7 ± 2.1	0.1664 ± 0.0014	13651 ± 126	13467 ± 181	422 ± 2	13400 ± 181
EC-16-5-F-30c	109.6 ± 0.2	164 ± 10	3912 ± 236	116.4 ± 2.0	0.3560 ± 0.0052	41576 ± 744	41537 ± 745	131 ± 2	41470 ± 745
EC-16-5-F-38b	242.2 ± 0.4	707 ± 18	1994 ± 55	108.8 ± 2.0	0.3532 ± 0.0033	41534 ± 483	41473 ± 485	122 ± 2	41406 ± 485
EC-16-5-F-48e	121.8 ± 0.2	741 ± 15	1005 ± 21	158.1 ± 1.9	0.3710 ± 0.0018	41718 ± 254	41568 ± 275	178 ± 2	41501 ± 275
EC-16-5-F-50z	104.9 ± 0.1	828 ± 20	795 ± 24	158.0 ± 2.2	0.3808 ± 0.0068	43055 ± 940	42566 ± 997	178 ± 3	42499 ± 997
EC-16-5-F-58z	109.7 ± 0.1	196 ± 8	3457 ± 148	148.3 ± 2.1	0.3752 ± 0.0045	42743 ± 628	42699 ± 628	167 ± 2	42632 ± 628
EC-16-5-F-68	198.2 ± 0.3	179 ± 4	7090 ± 145	176.6 ± 1.8	0.3878 ± 0.0012	43133 ± 183	43111 ± 183	199 ± 2	43045 ± 183
EC-16-5-F-73	143.5 ± 0.2	88 ± 2	10371 ± 216	106.7 ± 1.9	0.3853 ± 0.0012	46318 ± 208	46302 ± 209	122 ± 2	46236 ± 209
EC-16-5-F-80b	127.1 ± 0.2	275 ± 6	3043 ± 72	124.0 ± 2.0	0.3994 ± 0.0022	47449 ± 339	47034 ± 447	142 ± 2	46967 ± 447
EC-16-5-F-88	164.8 ± 0.2	456 ± 9	2474 ± 53	168.6 ± 1.8	0.4149 ± 0.0024	47266 ± 353	47199 ± 356	193 ± 2	47132 ± 356
EC-16-5-F-92d	129.2 ± 0.2	411 ± 9	2543 ± 56	260.0 ± 2.0	0.4907 ± 0.0025	52783 ± 362	51923 ± 706	301 ± 2	51856 ± 706
EC-16-5-F-116	68.6 ± 0.1	57 ± 3	9638 ± 480	255.2 ± 2.0	0.4895 ± 0.0031	52894 ± 437	52875 ± 437	296 ± 2	52808 ± 437
EC-16-5-F-127	83.9 ± 0.1	415 ± 9	1667 ± 35	253.5 ± 2.1	0.4996 ± 0.0022	54384 ± 325	53903 ± 469	295 ± 2	53836 ± 469
EC-16-5-F-135	109.1 ± 0.1	474 ± 10	1864 ± 39	237.0 ± 1.7	0.4914 ± 0.0020	54212 ± 296	54113 ± 304	276 ± 2	54046 ± 304
EC-16-5-F-140	126.8 ± 0.1	1066 ± 22	1037 ± 21	262.3 ± 1.9	0.5288 ± 0.0022	57945 ± 341	57758 ± 365	309 ± 2	57691 ± 365
EC-16-5-F-163	101.9 ± 0.1	2235 ± 45	422 ± 9	301.4 ± 2.1	0.5615 ± 0.0017	60061 ± 268	59589 ± 427	357 ± 2	59523 ± 427
EC-16-5-F-167b	125.4 ± 0.2	122 ± 3	10126 ± 264	180.3 ± 1.9	0.5972 ± 0.0019	75215 ± 386	75192 ± 386	223 ± 2	75125 ± 386

The error is 2σ error. U decay constants: λ_238_ = 1.55125 × 10^−10^ ^46^ and λ_234_ = 2.82206 × 10^−6^ ^47^. Th decay constant: λ_230_ = 9.1705 × 10^−6^ ^47^. *δ^234^U = ([^234^U/^238^U]activity – 1) × 1000. ^Ɨ^δ^234^U_initial_ was calculated based on ^230^Th age (T), i.e., δ^234^U_initial_ = δ^234^U_measured_ × e^λ234xT^. Corrected ^230^Th ages assume the initial ^230^Th/^232^Th atomic ratio of 4.4 ± 2.2 × 10^−6^. Those are the values for a material at secular equilibrium, with the bulk earth ^232^Th/^238^U value of 3.8. The errors are arbitrarily assumed to be 50%. ***BP stands for “Before Present” where the “Present” is defined as the year 1950 A.D.

### Stable isotopes

Stable isotope values range from −8.3 to −1.9‰ for δ^13^C and from −10.2 to −7.4‰ for δ^18^O. There is a statistically insignificant correlation between δ^18^O and δ^13^C during the last glacial period (R^2^ = 0.12), suggesting there are no major kinetic isotope effects during calcite precipitation^22^.

## Discussion

### Speleothem deposition at El Capitan Cave

Deposition of speleothem EC-16-5-F occurred in stepwise fashion, growing at ~0.02 mm/yr within each growth segment (Fig. 2). As no sulfides are present in the host rock to allow for sulfuric acid dissolution of the limestone in the absence of soils^23^, speleothem growth at this site most likely took place when soil was present in the catchment, thereby promoting the production of soil carbon dioxide and carbonic acid dissolution of the carbonate bedrock. It is likely that permafrost, had it existed, would have effectively shut down the infiltration of water and therefore prevented calcite deposition. The growth segments recorded in the stalagmite therefore most likely represent intervals of relatively mild climate when MAAT was above freezing.

The intervals of non-deposition range from 1,000 to 28,000 years in duration (Fig. 2). In total, six distinct hiatuses and one cryptic hiatus (Fig. 2) define the boundaries of the growth segments. The latter occurs between 59,300 ± 787 and 57,970 ± 777 yr BP. Upon visual inspection, this growth segment appears continuous (Fig. 2); however, oxygen and carbon isotope values change sharply (by 0.4‰ and 3.9‰, respectively) at 128.5 mm. If we assume continuous deposition between the two dated samples, stalagmite growth slowed considerably to 0.004 mm/yr, inconsistent with typical values of ~0.02 mm/yr. We therefore suggest a cryptic hiatus at 128.5 mm (Fig. 2). Applying the growth rate of 0.02 mm/yr, a period of growth occurred for ~450 years between 59,750 ± 421 and 59,300 ± 787 yr BP. The cryptic hiatus after 59,300 ± 840 yr BP spans ~1,300 yr. Growth re-commenced between 58,000 ± 782 and 57,596 ± 797 yr BP (Fig. 2).

The hiatuses recorded in speleothem EC-16-5-F could represent hydrological re-routing or blockage, as noted in speleothem studies elsewhere^24,25^. However, these processes tend to be permanent^26^, and are therefore not consistent with the continued growth of speleothem EC-16-5-F after each hiatus. Alternatively, periodic flooding of the cave could produce the observed hiatuses. However, there is no evidence of high-water marks or fine-grained slackwater deposits in the passageway where the speleothem was collected, or such detrital materials in the speleothem itself. Hiatuses could also form as a result of climate changes such as increased aridity^27^ or permafrost, preventing water flow and calcite deposition^28^. As the MAAT at the site is presently about 5 °C, it is plausible that the MAAT during stadials was at or below 0 °C, and may have resulted in the development of discontinuous permafrost. Temperatures within the cave were also likely to be at or below 0 °C, as interior cave air temperatures closely correspond to the MAAT. A drop of 5 °C has been reported in Pacific marine cores during interstadial/stadial transitions^9,11^. Glaciation may have also been a contributing factor in the formation of hiatuses by removing soils important for carbonate dissolution and speleothem deposition. However, the study of the glacial history in southeast Alaska/northern coastal British Columbia prior to the Last Glacial Maximum is still in its infancy. No glacial records in the region currently exist beyond the Last Glacial Maximum, which occurred between 17,000 to 22,000 yr BP^29^. Because of the uncertainty of glacial advances in the region, we prefer to use a more conservative approach and consider permafrost development to be a principal mechanism for hiatus formation in EC-16-5-F. U-Th ages constraining the individual growth phases and the intervening hiatuses are thus used to establish a record of mild and cold climate episodes in SE Alaska.

### Synchronicity between high-latitude North Pacific and North Atlantic regions

Currently there are no well-dated continuous terrestrial records from high-latitude sites in the North Pacific region that span the full interval of MIS 3, when D-O cycles are most prominent. In order to attempt to determine any apparent synchronicity between high-latitude North Pacific and North Atlantic regions, we compare episodic pulses of growth from speleothem EC-16-5-F with Greenland ice-core data.

**Figure 3:**
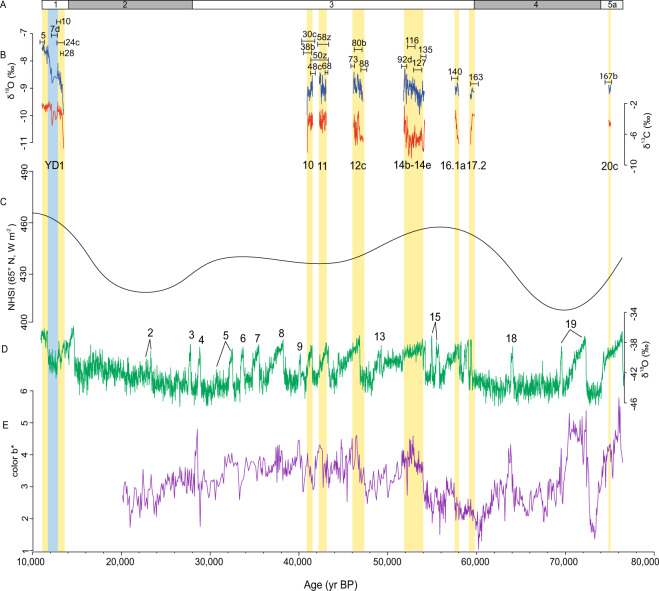
(**A**) Timing of Marine Isotope Stages 5a to 1^45^. (**B**) El Capitan Cave speleothem EC-16-5-F carbon and oxygen isotope record. Black bars show 2σ errors of the speleothem U-Th ages with corresponding sample numbers in Table 1. (**C**) NHSI (Northern Hemisphere summer insolation, 21 July) at 65°N^35^. (**D**) NGRIP oxygen isotope record^30^. (**E**) Color B* spectral analyses from core S0201-2-85KL^12^. Yellow vertical bars are used to highlight speleothem EC-16-5-F growth intervals.

The Greenland ice-core record serves as a Northern Hemisphere reference for alternating warm (Greenland interstadials, GI) and cold (Greenland stadials, GS) events during the last glacial period. We use the NGRIP record and the spliced GICCO5/ss09sea age model^30,31^ and follow the INTIMATE event stratigraphy^32^ (Fig. 3) in our comparison with the growth record of speleothem EC-16-5-F.

Growth phases recorded in speleothem EC-16-5-F are synchronous (within 2σ error of the time-depth model) with GI events^32^ (Fig. 3) providing strong evidence for D-O cycles in Alaska. However, difference between the records are also evident. Differences are initially seen in the termination of growth episodes in EC-16-5-F, marked by the presence of hiatuses. The termination of growth in EC-16-5-F is not always synchronous with GS events^32^. Calcite deposition at the end of GI-16.1a, GI-14b, and GI-12c stopped prior to the onset of the subsequent stadials in the Greenland record^32^ (Fig. 3). The modest discrepancies between records could possibly be due to permafrost sensitivity in Southeastern Alaska. Permafrost in the region may have developed with just a slight drop in MAAT, halting speleothem growth. This slight drop in temperature may only be subtle in the Greenland record, precluding it from being designated a GS event, but could have been significant enough to initiate the development of permafrost, resulting in the aforementioned hiatuses in EC-16-5-F. This demonstrates the high sensitivity of speleothem EC-16-5-F to changes in the MAAT, through the hypothesized linkage to permafrost development.

GIs 2*–*9, 13, 15, 18, and 19 are not recorded by speleothem EC-16-5-F. GI-13 has been reported as a minor interstadial in Greenland^30–32^. It is also poorly developed in the stalagmite records from Kleegruben Cave in the Austrian Alps^33^ and in Crag Cave in Ireland^34^, sites which reflect the North Atlantic climate. GI-15, represented by GI-15.1 and GI-15.2, may also reflect minor interstadials, with GI-15.1 only being only 100 years long^32^. The absence of GI′s 2*–*9, 18, and 19 in our speleothem record may reflect lower summer insolation during this overall time frame^35^ (Fig. 3). It is possible that permafrost persisted through these missing GI events in Southeastern Alaska. Permafrost has many complex interactions with the surrounding landscape, involving slope, aspect, soil conditions, vegetation, and snow cover, which can allow it to persist at MAATs as high as 2 °C^36^. However, even with the complexities of permafrost, the overall evidence suggests synchronicity between the high-latitude North Pacific and North Atlantic climates.

The atmospheric teleconnection observed between the Greenland record and speleothem EC-16-5-F is also observed in oceanic processes in marine core S0201-2-85KL in the western Bering Sea^12^. The marine core has a similar high-latitude as El Capitan cave at 57°30.30′N. The proxy used in core S0201-2-85KL as an age control beyond ~30,000 yr BP is color B* spectral analyses (sediment color analyses used as a proxy for diatom content vs. detrital input)^12^, in part due to its similar fluctuations with the NGRIP δ^18^O record^30^ (Fig. 3). Increasing color B* indicates more diatoms^37^, and in core SO201-2-85KL are considered brief intervals of enhanced marine productivity, sudden sea-ice melt associated with the subsequent release of ice-rafted debris, and a higher bottom water calcite saturation state, and may be related to D-O cycles observed in the NGRIP record^12^. Color B* spectral analyses was therefore chosen as the principle proxy to correlate with growth phases recorded in speleothem EC-16-5-F to determine if they were synchronous (Fig. 3).

Growth phases in speleothem EC-16-5-F are synchronous, within 2σ error, with increases in color B* during Greenland interstadial (GI) events 10, 11, 12c, 14b*–*e, and 20c^32^ (Fig. 3). GI events 16.1a and 17.2^32^, which are observed in speleothem EC-16-5-F, and are notable peaks in the Greenland INTIMATE chronology^32^ (Fig. 3), are not prominent in color B* in core S0201-2-85KL^12^. Color B* may not be a suitable proxy to show these events. Alternatively, there may be an error with wiggle-matching color B* in core S0201-2-85KL with the NGRIP record, or these two events are not well-expressed in all North Pacific marine records.

### Interpretation of stable isotopes

The episodic growth of speleothem EC-16-5-F prevents a continuous paleoclimate reconstruction; however, certain interpretations can still be made. During MIS 3, average δ^13^C and δ^18^O values are relatively stable, ranging from −6.5‰ to −4.2‰ and −9.2‰ to −8.9‰, respectively. Since growth periods are attributed to relatively mild conditions when permafrost is absent, large isotopic transitions leading into and out of stadials are mostly missing in the EC-16-5-F record.

The largest change in both δ^13^C and δ^18^O occurs at the end of GI*-*10, at 40,972 ± 831 yr BP, when δ^13^C and δ^18^O values decrease by 5.2‰ and 1.6‰, respectively (Fig. 3). This transition offers a glimpse of isotopic changes during a D-O cycle from an interstadial into a stadial. These isotopic shifts are difficult to interpret because no modern speleothem records exist for this region that would allow a calibration of the speleothem isotopic response to modern temperature/precipitation and vegetation changes. However, as moisture sources are not expected to have changed fundamentally during these periods, the large drop in δ^18^O most likely reflects cooler temperatures and/or an increase in the ratio of winter to summer precipitation. It is less clear why δ^13^C values decrease at this time. Cooling reflected in low δ^18^O values sometimes results in less soil productivity and higher δ^13^C values, but this is not the case in this record, which shows lower δ^18^O values corresponding to lower δ^13^C values (Fig. 3). A change in the vegetation, such as a change from gymnosperms to angiosperms^38^, instead of just soil productivity, could be responsible for this pattern. Gymnosperms are known to have enriched δ^13^C_leaf_ values compared to co-occurring angiosperms^39,40^. Furthermore, δ^13^C of bulk sediment from a lake core collected on Baker Island, 100 km south of El Capitan Cave, when compared to pollen from the same horizon, shows a clear trend toward lower δ^13^C values corresponding to increased angiosperm presence (*Alnus*) and occurs during the coldest part of the record during the Younger Dryas interval. Higher δ^13^C values correspond to increased gymnosperm presence (e.g. *Pinus, Tsuga mertensiana*) and occur during the warmer parts of the record^38^. Therefore, the area may have been covered by angiosperm herbs and shrubs during cold intervals, while gymnosperm woodlands expanded during warmer intervals. These changes in δ^18^O and δ^13^C may be related to shifts and/or strength of the Aleutian Low, with an enhanced Aleutian Low providing warmer/wetter conditions needed for speleothem growth at these northern latitudes.

### The Younger Dryas

It is interesting that the Younger Dryas chronozone (YD) is the only stadial fully represented in speleothem EC-16-5-F (Fig. 3). Although the rate of calcite deposition during this interval did slow somewhat, deposition was maintained during this stadial, and implies that permafrost and glaciation were largely absent. It is also interesting that isotope values follow the same trend as from the lake core collected on Baker Island *–* lower δ^13^C values during progressively colder time intervals. Growth of speleothem EC-16-5-F during the YD stadial may imply that cooling associated with the stadial may have been relatively subdued compared to other stadials described in the INTIMATE event stratigraphy^32^; however, this will require additional study.

### Summary

The episodic growth pattern of stalagmite EC-16-5-F from Southeastern Alaska represents a sensitive response to climate and new insight into the impacts of D-O events in the North Pacific realm. The independent chronology, when compared with a the Greenland ice core and North Pacific marine core records, provides strong evidence of teleconnections between the North Atlantic and North Pacific realms during MIS 3. Small differences in the timing of speleothem growth phases in climate-sensitive cave sites compared with the North Pacific, such as at GI 16.1a and 17.2, highlight the need for a network of studies using high-precision archives to map out the regional impact of D-O events.

## Methods

Speleothem EC-16-5-F was cut in half and polished. The orientation of the main growth axis changed several times in the speleothem during its growth (Fig. 2). Total length was measured at 140.5 mm. Thin sections were made and examined using optical microscopy, including blue-light epifluorescence.

A total of 21 powdered calcite samples were manually drilled for U-Th dating under a laminar flow hood. An additional 452 samples were collected for stable isotope analyses using a Merchantek micromill every 0.3 mm from 0 to 133.5 mm and every 1 mm from 133.5 to 140.5 mm. All distances are measured from the top of the stalagmite (Fig. 2).

U-Th samples were processed at the University of Minnesota Trace Metal Isotope Geochemistry Lab and analyzed using a ThermoFisher Neptune Plus multi-collector inductively coupled plasma mass spectrometer equipped with an Aridus desolvation nebuliser, following the method of Shen *et al*.^41^. Ages are reported with 2σ errors in years before present, i.e. before the year 1950 A.D. A time-depth model was created in OxCal 4.3 using the Bayesian approach^42,43^, with a variable “k” parameter of 0.1–0.2 mm^−1^ (Fig. 2). The duration of the hiatuses were estimated using the time-depth model (Fig. 2).

Stable isotope samples were analyzed at the University of Innsbruck using a ThermoFisher Delta V isotope ratio mass spectrometer equipped with a Gasbench II^44^. Stable isotopes are reported in per mil relative to Vienna Peedee Belemnite (VPDB). Long-term analytical precision is better than 0.08‰ for both δ^13^C and δ^18^O (1 σ). The stable isotopes were incorporated into the OxCal 4.3 time-depth model.

## Supplementary information

Petrographic observations of hiatuses

## Data Availability

https://www.ncdc.noaa.gov/paleo/study/26770.
