# Author Correction: Millennial-scale glacial climate variability in Southeastern Alaska follows Dansgaard-Oeschger cyclicity

**DOI:** 10.1038/s41598-023-29698-3

**Published:** 2023-02-21

**Authors:** Paul S. Wilcox, Jeffrey A. Dorale, James F. Baichtal, Christoph Spötl, Sarah J. Fowell, R. Lawrence Edwards, Johanna L. Kovarik

**Affiliations:** 1 Geoscience Department, University of Alaska Fairbanks, Fairbanks, AK 99775 USA; 2 Institute of Geology, University of Innsbruck, 6020 Innsbruck, Austria; 3 Department of Earth and Environmental Sciences, University of Iowa, Iowa City, IA 52252 USA; 4 Forest Service, Tongass National Forest, Thorne Bay, AK 99919 USA; 5 Department of Earth Sciences, University of Minnesota, Minneapolis, MN 55455 USA; 6 Forest Service, Mineral and Geology Management, Denver, Colorado 80401 USA

Correction to: *Scientific Reports* 10.1038/s41598-019-44231-1, published online 27 May 2019

This Article contains errors in the Results and Discussion sections as well as in Figure 3.

Firstly, in the Results section, under the subheading ‘Chronology’, where the last growth phase estimated from the time-depth model is incorrectly given as “between 13,602 ± 459 and 11,100 ± 598 yr BP”. The correct phase should read “between 13,633 ± 543 and 11,280 ± 821 yr BP”. Furthermore, the following sentence should be deleted: “The growth period between 13,602 ± 459 and 11,100 ± 598 yr BP records the Younger Dryas interval.”

Secondly, in the Discussion section, under the subheading ‘The Younger Dryas’, the following sentences should be deleted: “Although the rate of calcite deposition during this interval did slow somewhat, deposition was maintained during this stadial, and implies that permafrost and glaciation were largely absent. It is also interesting that isotope values follow the same trend as from the lake core collected on Baker Island—lower δ^13^C values during progressively colder time intervals.”

Finally, in Figure 3b the blue and red lines representing the El Capitan Cave speleothem EC-16-5-F carbon and oxygen isotope record are incorrect. The correct Figure 3 and accompanying legend appear below.

**Figure 3:**
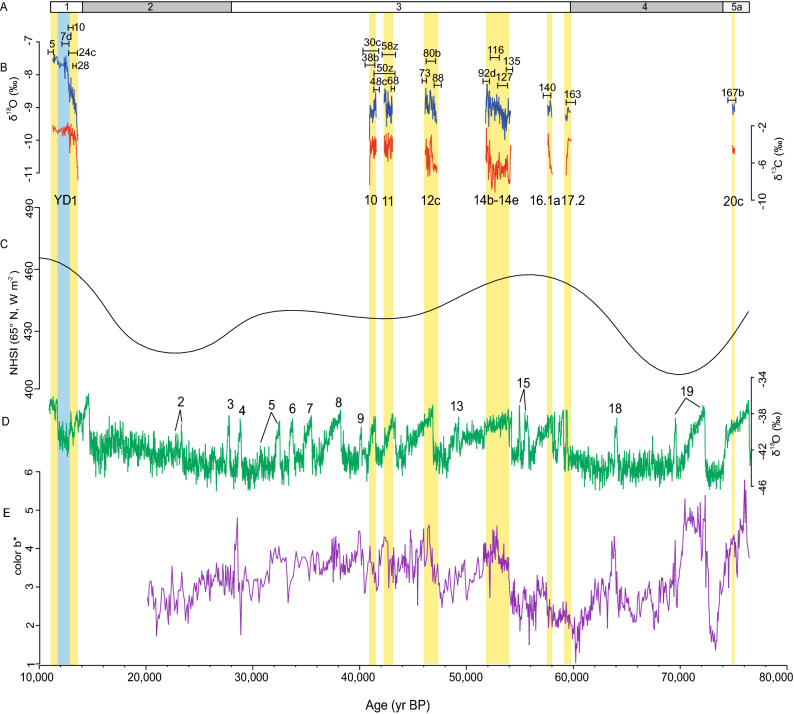
(**A**) Timing of Marine Isotope Stages 5a to 1^45^. (**B**) El Capitan Cave speleothem EC-16-5-F carbon and oxygen isotope record. Black bars show 2σ errors of the speleothem U-Th ages with corresponding sample numbers in Table 1. (**C**) NHSI (Northern Hemisphere summer insolation, 21 July) at 65°N^35^. (**D**) NGRIP oxygen isotope record^30^. (**E**) Color B* spectral analyses from core S0201-2-85KL^12^. Yellow vertical bars are used to highlight speleothem EC-16-5-F growth intervals.

