# Supplementary material for: Millennial-scale glacial climate variability in Southeastern Alaska follows Dansgaard-Oeschger cyclicity
Source: Sci Rep. 2019 May 27;9:7880. doi: 10.1038/s41598-019-44231-1 (PMC6536552; doi:10.1038/s41598-019-44231-1)
Supplement: Supplementary file 1 — Petrographic observations of hiatuses [file 41598_2019_44231_MOESM1_ESM.pdf]

**Supplement 1:** Petrographic observations of hiatuses
**Tile of manuscript:** Millennial-scale glacial climate variability in Southeastern Alaska follows Dansgaard-Oeschger cyclicity
**Authors**: Paul S. Wilcox, Jeffrey A. Dorale, James F. Baichtal, Christoph Spötl, Sarah J. Fowell, R. Lawrence Edwards, Johanna L. Kovarik

**Description**: The six hiatuses identified macroscopically in speleothem EC-16-5-F are also prominently seen in thin sections. Figures S1 and S2 provide triplets of photomicrographs documenting the petrographic expression of growth stops under polarized light and under epifluorescence.

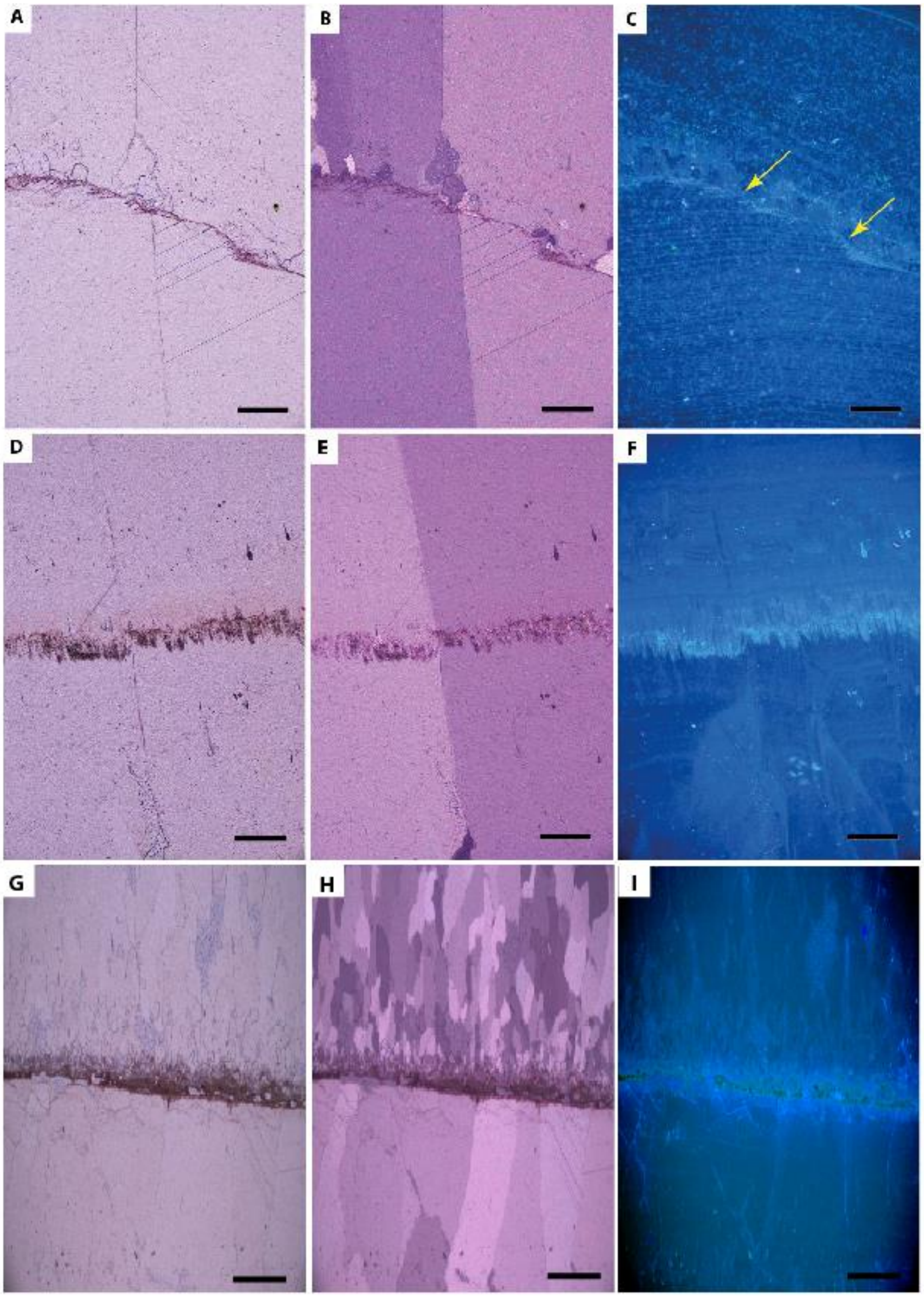


Fig. S1: A-C: Thin-section photomicrographs of the youngest hiatus at 27.9 mm distance from top. Note dissolution at this boundary as seen by the abrupt termination of regular growth laminae visualized by epifluorescence (arrows in C), as well as partial re-nucleation of columnar calcite crystals at this boundary. D-F: Photomicrographs of the hiatus at 45.9 mm distance from top marked by a micritic layer. Note continuity of the large columnar crystals across the boundary and no evidence of corrosion or re-nucleation of calcite crystals. G-I: Photomicrographs of the hiatus at 69 mm distance from top showing termination of the older coarsely crystalline calcite and re-start of crystal growth afterwards.

A, D, G: transmitted-light, parallel nicols, B, E, H: transmitted-light, partially crossed nicols, C, F, I: blue-light epifluorescence. Growth direction is up on all images. Scale bar 0.2 mm.

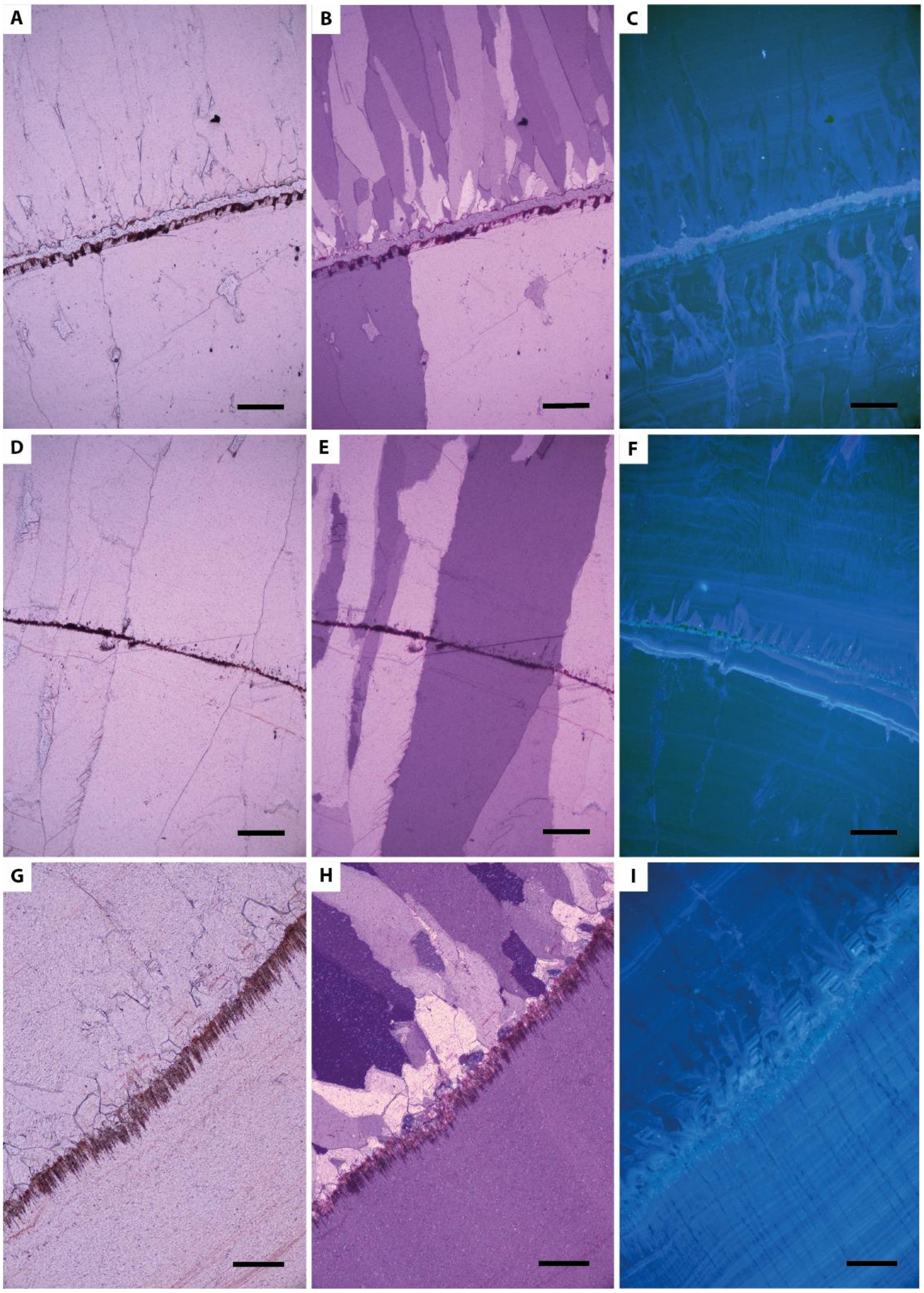


Fig. S2: A-C: Thin-section photomicrographs of the hiatus at 84.9 mm distance from top showing termination of the older coarsely crystalline calcite and re-start of crystal growth afterwards. The crack at the hiatus in an artifact of thin-section preparation. D-F: Photomicrographs of the hiatus at 129.6 mm distance from top. While most columnar calcite crystals cross this boundary, some crystal re-nucleation occurred at this hiatus as shown by the epifluorescence image (F). G-I: Photomicrographs of the deepest hiatus at 139.3 mm distance from top. Crystal tips are lined by micrite and none of the previous columnar crystals crosses this boundary. Note regular laminae in both the pre- and post-hiatus calcite under epifluorescence (I). A, D, G: transmitted-light, parallel nicols, B, E. H: transmitted-light, partially crossed nicols, C, F, I: blue-light epifluorescence. Growth direction is up on all images. Scale bar is 0.5 mm in A-F and 0.2 mm in G-I.
